# The Fourth Ventricle, A Rare Location for Subependymal Giant Cell Astrocytoma (SEGA): A Rare Case Report

**DOI:** 10.1002/ccr3.71705

**Published:** 2025-12-19

**Authors:** Ali Naeim, Mohammad Reza Hosseinkazemi, Ali Fakih, Yalda Nilipour, Peyman Gookizadeh

**Affiliations:** ^1^ Department of Neurosurgery Sina Hospital, Tehran University of Medical Sciences Tehran Iran; ^2^ Department of Pathology Toos Hospital, Shahid Beheshti University of Medical Sciences Tehran Iran

**Keywords:** case report, fourth ventricle, subependymal giant cell astrocytoma, tuberous sclerosis

## Abstract

Subependymal giant cell astrocytoma (SEGA) is classified as a WHO grade I tumor. This tumor is often detected in the lateral ventricles and accompanies tuberous sclerosis complex (TSC). Here, we report a patient with SEGA in the fourth ventricle for the first time.

## Graphical abstract

Preoperative MRI of a SEGA lesion in the fourth ventricle.

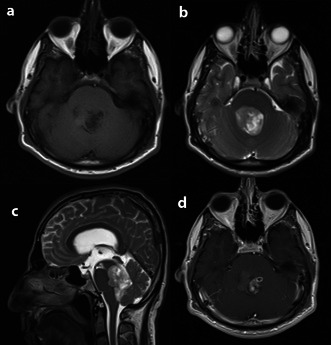

## 1 Introduction

Subependymal giant cell astrocytoma (SEGA) is a benign intraventricular tumor [1] and the most common central nervous system lesion in patients with tuberous sclerosis complex (TSC). It is often found near the Foramen of Monro and may present with chronic hydrocephalus symptoms like headaches, vision changes, seizures, or gait disturbances [2, 3]. However, SEGA is a characteristic lesion of TSC; a few cases of solitary, non‐TSC SEGA have been reported [4]. To our knowledge, there has not been any report of a SEGA lesion located in the fourth ventricle. In this article, we present a case of clinically non‐TSC SEGA lesion in the fourth ventricle for the first time, review the literature and introduce SEGA as a possible fourth ventricle lesion.

## 2 Case History

A 40‐year‐old male patient was referred with a history of dizziness over several months associated with nausea and vomiting and mild generalized headache during the previous two months.

He had no history of medical issues or medication use. In his examination, he had stable vital signs and no neurological or cognitive status impairment was detected. Ophthalmologic examination revealed papilledema graded II on the Frisen scale. The patient had ataxic gait, but there were no motor or sensory neurological deficits in the extremities. Cranial nerve examination and reflexes were intact. All routine lab tests showed normal values.

**FIGURE 1:**
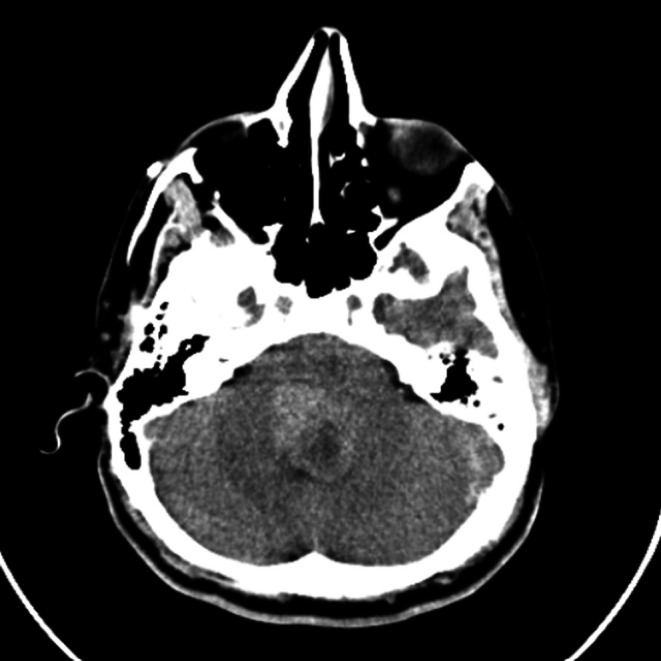
Axial plane of the patient's brain CT scan showing a heterogeneous hyperdense lesion located in the fourth ventricle.

His brain CT showed a heterogeneous hyperdense lesion, located in the fourth ventricle, causing it to be fully obliterated. Also, we saw obstructive hydrocephalus in the 3rd and lateral ventricles along with partial gyrus effacement without periventricular edema (Figure 1).

**FIGURE 2:**
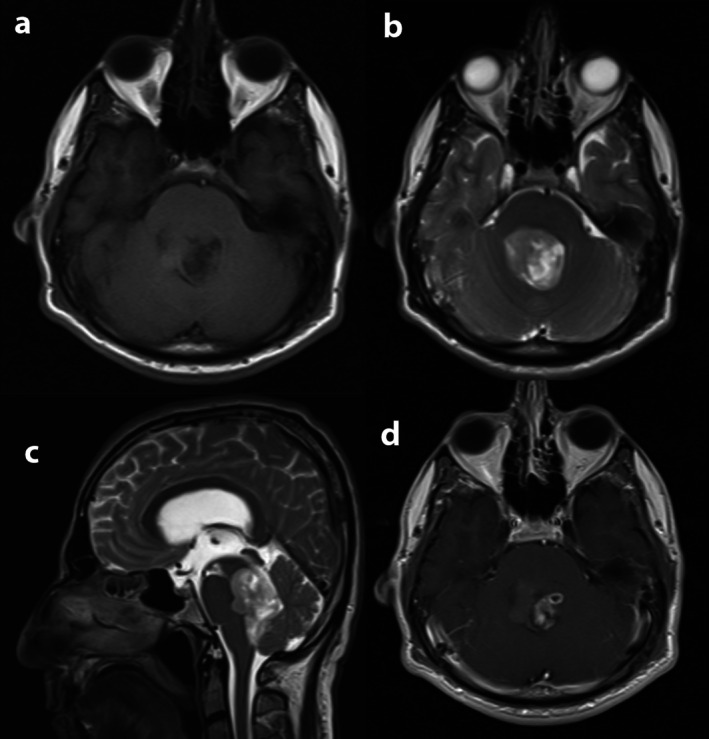
Preoperative MRI: (a) Axial T1W. (b) Axial T2W. (c) Sagittal T2W. (d) T1W with gadolinium.

The brain MRI revealed a solid‐cystic intraventricular lesion measuring 35 × 30 at maximum diameter, T1‐weighted iso to hyposignal, T2‐weighted hypersignal and heterogeneous enhancement after gadolinium injection, located in the fourth ventricle with mass effect on brainstem's posterior side and vermis (Figure 2).

## 3 Methods

The patient underwent a midline suboccipital craniotomy and a gross total resection of the fourth ventricular tumor was achieved through a Telovelar approach with right keen's point EVD insertion for CSF diversion.

## 4 Conclusions and Results

Postoperative, the patient developed worsening gait disturbance, left abducens nerve palsy causing esotropia, and facial nerve palsy with a House‐Brackmann score of IV. In addition, the patient showed a recovery from hydrocephalus which was also expressed in his postoperative brain CT scan.

**FIGURE 3:**
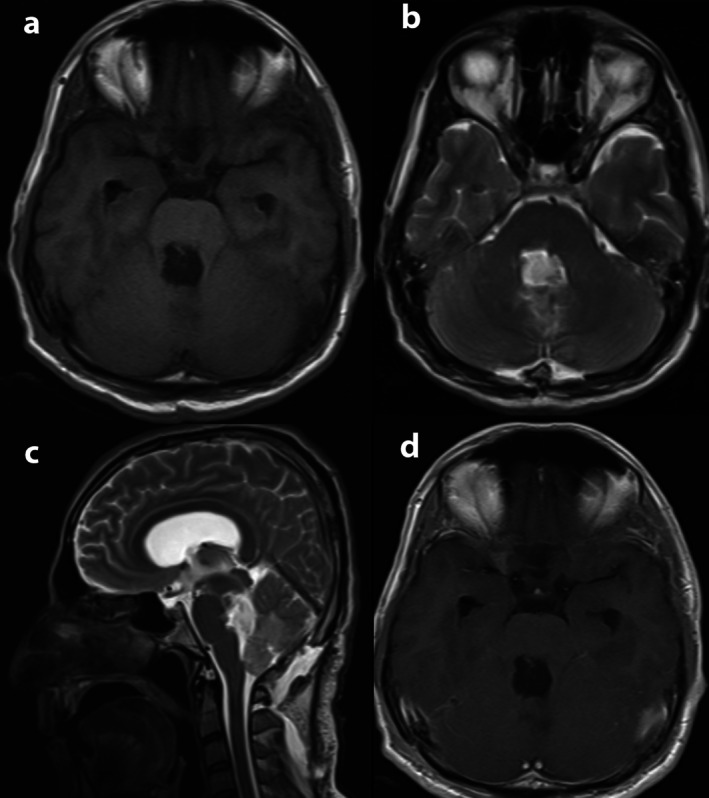
Postoperative MRI: (a) Axial T1W. (b) Axial T2W. (c) Sagittal T2W. (d) T1W with gadolinium.

Post‐op brain MRI showed some postop changes after suboccipital craniectomy, resection of fourth ventricle mass with mild pneumocephalus and mild hemorrhagic content in the surgical bed, and ventriculostomy in the right lateral ventricle. No clear evidence of tumor remnant was seen (Figure 3).

**FIGURE 4:**
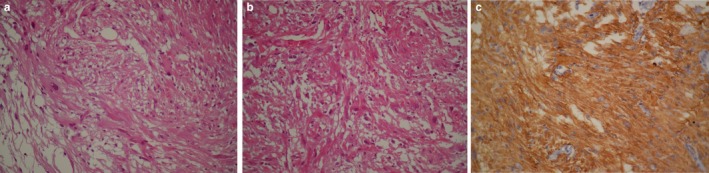
(a) Neoplastic proliferation of large spindle and polygonal cells with abundant eosinophilic cytoplasm and pleomorphic nuclei, some with pseudoinclusions, in sweeping fascicles and sheets with intervening fibrillary septa (H&E 400×). (b) Sheets of large pleomorphic gemistocyte‐or ganglionic‐like cells with some multinucleated forms (H&E 400×). (c) All tumor cells are positive for GFAP (immunohistochemistry 400×).

The surgical specimen was reported as multiple pieces of grayish tissue measuring 3.5 × 1 × 0.6 cm. Histopathological examination showed a moderately cellular neoplastic tissue composed of large spindle or polygonal cells with abundant glassy cytoplasm and nuclear pleomorphism with multinucleation arranged in sweeping fascicles with intervening fibrillary septa without necrosis or vascular proliferation (Figure 4a,b). For confirmation, immunohistochemical study was done, showing positive staining with TTF1, S100 and glial fibrillary acidic protein (GFAP) antibodies (Figure 4c) and negative staining for EMA and neuronal markers. The proliferation index was assessed with Ki67 immunomarker and was found to be about 2% in tumor cells. Based on the above histologic and immunohistochemical findings, the diagnosis of low‐grade astrocytoma in favor of SEGA was made. Although tumors like ependymoma or subependymoma are more frequently seen in this location, regarding typical histologic findings as large pleomorphic cells and absence of neither nuclear clustering nor microcystic change, accompanied by mentioned immunoprofile, the diagnosis of SEGA was confirmed.

The patient was examined retrospectively later, and neither major nor minor clinical features of the TSC were found.

## 5 Discussion

SEGA is a World Health Organization (WHO) Grade I slow‐growing tumor that typically originates in the walls of the lateral ventricles near the foramen of Monro and rarely in the third ventricle [1, 4]. It is usually presented in the first two decades of life and is often associated with TSC [5]. However, a few cases of SEGA without any clinical features of TSC have been reported [4, 6]. Due to its location, symptomatic SEGA lesions classically present with features of elevated intracranial pressure secondary to obstructive hydrocephalus, such as headache, papilledema, or seizures [2, 7].

In a 20‐case series report in 2017 by Mei, all of the cases originated in lateral ventricles; the literature review shows that SEGA is classically presented near foramen of Monro as well [1, 5, 6]. Our pathology reported SEGA in the fourth ventricle, and our literature review found that this is the first case which is presented with this pathology in the fourth ventricle. Since the fourth ventricle is a surgically challenging anatomical region for surgery, reporting SEGA as a low‐grade lesion for possible pathologies of this area could help with planning for treatment.

Shelley's study in 2023 reported 7 cases of clinically and genetically isolated SEGA lesions. One of these cases presented with the lesion in the occipital lobe [4]. Although we did not perform genetic testing in this case, the patient was retrospectively examined for minor or major clinical features of TSC and had none of them. This suggests that isolated cases could accompany rare locations to be investigated in future studies.

We aim to focus here on the appearance of this tumor in a very rare location that has not been reported in any other publication. While most other cases have reported SEGAs presenting in the lateral ventricle and rarely in the third ventricle, our case involved a patient without any characteristics of TSC, unveiling a SEGA tumor originating from the fourth ventricle.

The purpose of this study was to delineate another growth origin location for SEGA that has not been previously detected or is rarely reported, which will include SEGA tumors in the differential diagnosis of any mass near the fourth ventricle.

## Author Contributions

**Ali Naeim:** conceptualization, formal analysis, resources, supervision, writing – original draft. **Mohammad Reza Hosseinkazemi:** resources, visualization. **Ali Fakih:** data curation, methodology, writing – original draft. **Yalda Nilipour:** data curation, supervision, visualization. **Peyman Gookizadeh:** methodology, writing – original draft, writing – review and editing.

## Funding

The authors have nothing to report.

## Ethics Statement

This study did not require ethics committee approval according to local regulations.

## Consent

Written informed consent for publication of their clinical details and clinical images was obtained from the patient described in this study.

## Conflicts of Interest

The authors declare no conflicts of interest.

## Data Availability

The data that support the findings of this study are available on request from the corresponding author. The data are not publicly available due to privacy or ethical restrictions.
